# Evidence of Workplace Interventions—A Systematic Review of Systematic Reviews

**DOI:** 10.3390/ijerph16193553

**Published:** 2019-09-23

**Authors:** Claudia Pieper, Sarah Schröer, Anna-Lisa Eilerts

**Affiliations:** Institute for Medical Informatics, Biometry and Epidemiology, University Hospital of Essen, 45147 Essen, Germany; sarah.schroeer@uk-essen.de (S.S.); anna-lisa.eilerts@uk-essen.de (A.-L.E.)

**Keywords:** health promotion, occupational, workplace, absenteeism, health care costs, evidence

## Abstract

Work environment factors are highly correlated with employees’ health and well-being. Our aim was to sum up current evidence of health promotion interventions in the workplace, focusing on interventions for the prevention of musculoskeletal disorders, psychological and behavioral disorders as well as interventions for older employees and economic evaluations. We conducted a comprehensive literature search including systematic reviews published from April 2012 to October 2017 in electronic databases and search engines, websites of relevant organizations and institutions. It consisted of simple and specific terms and word combinations related to workplace health promotion based on the search strategy of a previous review. After full-text screening, 74 references met the eligibility criteria. Using the same search strategy, there was a higher proportion of relevant high-quality studies as compared with the earlier review. The heterogeneity of health promotion interventions regarding intervention components, settings and study populations still limits the comparability of studies. Future studies should also address the societal and insurer perspective, including costs to the worker such as lost income and lost time at work of family members due to caregiving activities. To this end, more high-quality evidence is needed.

## 1. Introduction

Work can be a source of psychological distress and ill health [1]. Improving working conditions may promote physical and mental health by combining both the individual and organizational level. A number of reviews and single studies have addressed the efficacy and cost-effectiveness of well-designed worksite health promotion programs to improve the health of employees and save money for employers [2,3,4,5,6,7,8,9,10,11,12,13].

Work environment factors including shift work, work stress and work demands are highly correlated with employees’ health and well-being [14,15,16]. Work-related diseases cause lost working days, increase absenteeism and presentism, and reduce productivity.

Chronic diseases are among the most common and costly of all health problems worldwide. In Germany in 2016, 154 million days of incapacity to work due to musculoskeletal disorders led to a loss of 17.2 billion Euros in production costs and a loss of 30.4 billion Euros in gross value added. With 109.2 million days of incapacity to work, psychological and behavioral disorders rank in second place according to the calculations of the Bundesanstalt für Arbeitsschutz und Arbeitsmedizin (BAuA) for 2016. Psychological and behavioral disorders led to a loss in production costs of 12.2 billion Euros and a loss of gross value added of 21.5 billion Euros [17].

Promoting employees’ health therefore is of great importance for organizations as well as employees to maintain performance capability and productivity. However, does the current evidence of workplace health promotion and prevention encourage employers to provide their employees with preventive programs, training and tools that support healthy behaviors?

In 2014 our working group published a systematic review by order of the “Initiative Gesundheit und Arbeit (IGA)” that summarized the evidence of effectiveness of workplace health promotion and prevention, including publications from 2006 to 2012 [18,19]. Most reviews included studies of different intervention types focusing on physical activity, nutrition or weight management, alcohol dependence and tobacco use, as well as mental health and stress. Because of the heterogeneity of interventions, target groups and study designs, the overall evidence of effectiveness for the respective interventions was no more than moderate. Altogether, the findings emphasize the advantages of complex multi-component interventions. However, it could not be shown which approach leads to the best economic and health-related results for certain occupational groups. 

To determine whether there are any new findings, we aimed to synthesize evidence on the (cost) effectiveness of different workplace interventions from recently published systematic reviews (2012–2017). Our current systematic review was designed to provide evidence-based answers on key questions related to common health problems in the workplace and to offer information on available evidence across occupational groups. Effectiveness was determined by regarding the intervention’s impact on health-related outcomes, work disability duration, quality of life and others as well as economic outcomes. Interventions were defined as planned intervention programs in companies.

Because of the impact of musculoskeletal disorders, psychological and behavioral disorders and the proportion of older employees in Germany [20,21,22,23,24], we focused on interventions for the prevention of musculoskeletal, psychological and behavioral disorders as well as interventions for older employees. Additionally, we involved economic evaluations.

Our main research questions were:

Which evidence-based recommendations can be derived,

1. for the prevention of musculoskeletal diseases;
2. for the prevention of mental disorders;
3. for the strengthening of older employees;
4. on the economic impact of workplace interventions?

## 2. Methods

### 2.1. Search Process

We conducted a comprehensive literature from October to December 2017 including systematic reviews published from April 2012 to October 2017 in five electronic databases and search engines (PubMed, Cochrane Library, London, UK, Scopus, Web of Science and PsycINFO). In addition, the websites of relevant organizations and institutions were screened (German Bundesanstalt für Arbeitsschutz und Arbeitsmedizin BAuA, Dortmund, Germany, Institut für Arbeitsschutz der Deutschen Gesetzlichen Unfallversicherung, Sankt Augustin, Germany, Campell Collaboration, Oslo, Norway, International Labour Organization, Geneva, Switzerland, Partnership for European Research in Occupational Safety and Health, British Occupational Research Foundation, EPPI-Centre, Centre for Reviews and Dissemination and The National Institute for Occupational Safety and Health). We conducted an advanced search in the archives of the following journals: European Journal of Public Health, Journal Health Promotion International, Journal of Public Health, Oxford Economic Papers, Journal of Occupational and Environmental Medicine, American Journal of Preventive Medicine, Journal of Safety, Health and Environmental Research and Journal Work, Aging and Retirement. Reference lists of all retrieved articles were checked for further relevant publications. We limited our scope to systematic reviews published in peer-reviewed journals to maximize the validity of the findings and to reduce bias. All included publications were formally rated as systematic review articles. However, methodological quality did not always meet the Cochrane Collaboration quality criteria for systematic reviews. The language had to be English or German. The systematic literature search consisted of simple and specific terms and word combinations related to workplace health promotion based on the search strategy of Pieper et al. (2015) [18]. Combinations of search terms were run in all five databases. We used the following search string to identify relevant articles:

musculoskeletal diseases OR musculoskeletal pain OR musculoskeletal system/injuries OR musculoskeletal system/pathology OR musculoskeletal system/physiopathology.

OR

diseases category/psychology OR burnout, professional OR depression OR depressive disorder OR depressive disorder, major OR mental disorders OR mental illness.

OR

older workers OR elder workers OR aged workers OR aged employees OR elderly employees OR aged worker OR mature workers OR aging workforce OR aging working population OR aging employees OR aging employee.

AND

occupational health OR worksite health OR health promotion OR health prevention OR organisational health OR organizational health OR industrial health OR health interventions. 

AND 

effect OR impact OR cost allocation OR cost OR cost effect OR cost effective OR cost effective benefits OR cost effective disease management OR return on investment OR health economics OR health economics research OR health research OR health impact OR qalys OR quality adjusted life years OR absenteeism OR presenteeism OR cost benefit analysis OR cost effectiveness OR effectiveness OR health benefit plans, employee OR health care evaluation mechanisms OR evaluation OR efficiency OR benefit OR advantage.

### 2.2. Eligibility Criteria

The assessment of references was carried out in two phases, based on eligibility criteria described hereafter. In the first phase, all authors checked the titles and abstracts of the search results and reviewed the abstracts to determine whether to obtain the identified articles for a full-text search. Reviews were included in full-text search if the reported workplace interventions addressed health- and/or work-related outcomes in the prevention of musculoskeletal disorders, mental illnesses or the strengthening of older employees. Interventions were to focus on either individual, organizational, or combined-level health promotion or prevention at work. The study population included male and female employees in different age groups.

In the second phase, included full texts were finally assessed according to the selection criteria in detail, as comprehensible description of intervention effects and assessment of evidence. We developed a synthesis of included reviews and extracted the results reported in the reviews. Main outcomes were mean effect sizes and evidence levels. Ethical approval was not required, as the study was designed as a secondary literature review without human subjects, medical records or human tissues being directly involved.

### 2.3. Data Extraction and Study Quality Appraisal

In order to examine the quality of the finally included reviews, we extracted data source, database, study population, evaluated outcomes, reported effects and methodological aspects. Two authors independently analyzed the reviews using the AMSTAR 2 checklist [25]. Data were extracted by one author and checked by a second author. Discrepancies were resolved by consensus or by consulting a third person. AMSTAR is a critical appraisal tool for systematic reviews of randomized studies whereas AMSTAR 2 accounts for both randomized studies and observational (non-randomized) studies. Rating is based on weaknesses in critical domains:

- High—The systematic review provides an accurate and comprehensive summary of the results of the available studies that address the question of interest.
- Moderate—The systematic review has more than one weakness, but no critical flaws. It may provide an accurate summary of the results of the available studies that were included in the review.
- Low—The review has a critical flaw and may not provide an accurate and comprehensive summary of the available studies that address the question of interest.
- Critically low—The review has more than one critical flaw and should not be relied on to provide an accurate and comprehensive summary of the available studies.

## 3. Results

### 3.1. Search Results

**Figure 1:**
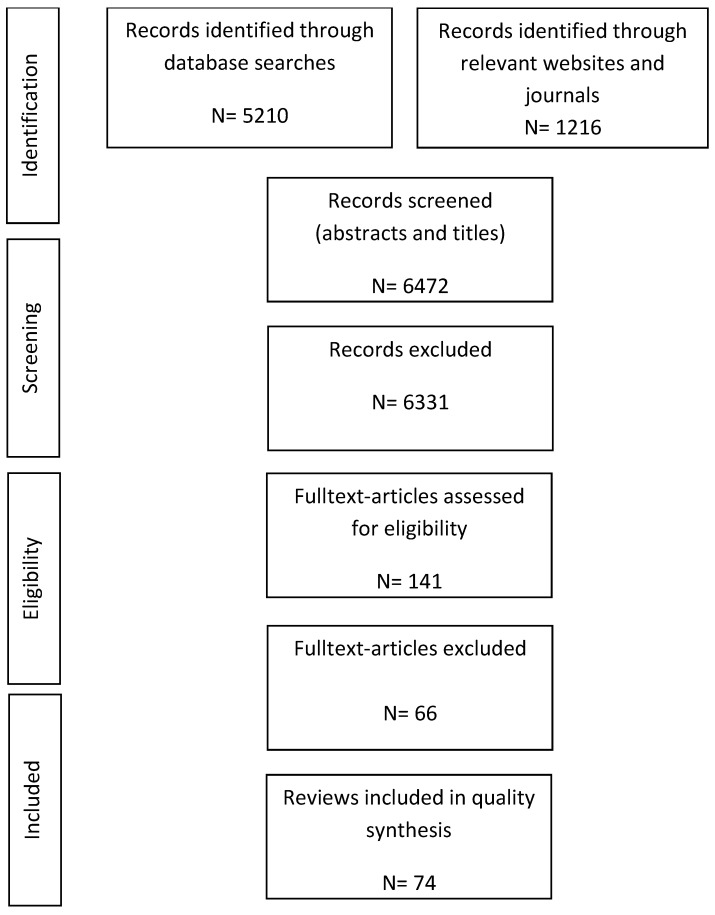
Flow chart of reviews included.

The original search identified 6472 references: PubMed *n* = 927, Scopus *n* = 291, Web of Science *n* = 1902, PsycINFO *n* = 566, Cochrane Library *n* = 1524, websites *n* = 1036 and topic-specific journals *n* = 226. Of these, 6331 references were rejected due to inappropriate outcome evaluation, inadequate study design or population. 141 reviews were included in the full-text screening. Finally, after full text screening, 74 references met the eligibility criteria. Figure 1 shows the results of the search process. 

These 74 reviews originated from the USA, Canada, Australia, New Zealand, and Europe, including 1715 single studies. The study designs were heterogeneous, 546 reviews (32%) included randomized controlled trials. Study populations comprised occupational groups from different sectors, including both men and women in varying percentages. 

### 3.2. Main Outcome Categories

We identified 38 reviews on the prevention and improvement of mental disorders. 23 reviews broached the issue of musculoskeletal disorders and four reviews reported interventions for older employees. Ten reviews reported economic outcomes of workplace interventions. Because one review refers to musculoskeletal disorders as well as to mental disorders, the total number of reviews is 74. Table 1 shows the distribution of reviews by quality rating and main outcome category. High and moderate-quality reviews were taken into account in the assessment of intervention efficacy. Low quality was due to the lack of information on interventions, confounders, dropout rates, and data analysis techniques.

Several systematic reviews of mental health and musculoskeletal disorders identified the same primary studies, depending on the search strategy, inclusion criteria and execution period. We will discuss in more detail below (see Section 4). Findings are presented according to quality of reviews and then according to interventions.

### 3.3. Mental Health

38 reviews on workplace interventions for the prevention and improvement of mental disorders were identified [2,4,6,7,12,13,26,27,28,29,30,31,32,33,34,35,36,37,38,39,40,41,42,43,44,45,46,47,48,49,50,51,52,53,54,55,56]. In these reviews, 1191 individual studies evaluated the effectiveness of resilience training programs, mindfulness training, cognitive-behavioral therapy, relaxation techniques and organizational-level workplace interventions. The focus of interventions varied, but those targeting specific individuals were outnumbered. Participants were predominantly white-collar workers, teachers and health care providers.

Seven high-quality reviews and one moderate-quality review found intervention effects on well-being, burn-out-symptoms and employees’ performance. Mindfulness and cognitive-behavioral training as well as peer supervision appeared to help reduce stress. Additionally, organizational interventions including reduction of work impact and flexible work-time seemed to lower stress and burn-out-symptoms. Overall, multi-component programs were more effective than single-component interventions. No reviews derived general recommendations. Few reviews recommended special interventions or intervention components with caution [7,42,44,46,50,54,56]. The authors found cognitive-behavioral effective to reduce depression, anxiety, and burnout as well as to improve well-being.

One moderate-quality review evaluated evidence for physical training and yoga-interventions, in which especially Yoga was found to be especially effective in the prevention of stress and anxiety [40].

Two moderate-quality reviews reported effectiveness of web-based health promotion programs for the prevention of job-stress related mental disorders [47,48]. Carolan, Harris and Cavanagh made a clear recommendation for implementation [47].

Cognitive-behavioral theories provided the main rationale for intervention programs in fourteen reviews [2,7,13,26,28,37,38,42,43,47,48,49,53,57]. Eight reviews addressed the prevention of mental disorders including depression, stress, and mental health [7,37,42,47,48,49,53,57]. Positive effects were shown for cognitive-behavioral interventions [2,7,26,28,37,42,47,49,57] and web-based cognitive-behavioral interventions [48]. Cognitive-behavioral interventions were effective to reduce depression, anxiety and burnout as well as to improve well-being [13,33,36]. Nevertheless, cognitive-behavioral interventions were not always superior to comparative interventions [7,26,47]. Results rarely reached statistical significance.

Mindfulness trainings was assessed in twenty reviews that found it to have beneficial effects on mental health including stress, anxiety, depression and burnout as well as well-being [4,12,13,28,30,31,35,36,39,43,44,45,46,47,48,50,53,54,55,56]. Results did not always reach statistical significance. Twelve reviews included mindfulness-based stress reduction (MBSR) interventions; ten reviews evaluated abbreviated MBSR interventions. Both MBSR and abbreviated MBSR were shown to have positive effects [12,35]. 

One moderate-quality review evaluated evidence for physical training and yoga-interventions, in which yoga was found to be especially effective in the prevention of stress and anxiety [40].

Two moderate-quality reviews reported effectiveness of web-based health promotion programs for the prevention of job-stress related mental disorders [47,48]. Carolan, Harris and Cavanagh made a clear recommendation for implementation [47].

### 3.4. Musculoskeletal System

23 reviews on workplace interventions for the prevention and improvement of musculoskeletal disorders were identified [3,5,8,9,10,11,34,58,59,60,61,62,63,64,65,66,67,68,69,70,71,72,73]. In these reviews, 407 individual studies were summarized, predominantly evaluating behavioral preventive interventions, such as training and education, exercise programs, and ergonomic interventions. The evaluated outcome parameters mainly referred to the upper musculoskeletal system, especially the back, neck and shoulders. In total, six reviews were rated as high-quality and eight reviews as moderate-quality.

One moderate-quality review found ergonomic interventions such as adjustable Sit to Stand Desks associated with reduction of neck or back pain [65], while two high-quality reviews did not [8,70]. Follow-up beyond a year was not conducted. Job rotation interventions resulted in opposing or no effects [63,69], while workplace strength training significantly reduced pain and prevented disorders in the upper musculoskeletal system, especially back, neck and shoulders [64].

Two low-quality reviews showed limited evidence for the effectiveness of physical activity programs.

An unrestricted recommendation cannot be made due to the heterogeneity of the studies included regarding investigated interventions, study populations and study designs. Overall, there was insufficient evidence to support the sustainability of reported effects beyond one year.

### 3.5. Older Employees

Four moderate-quality reviews on workplace interventions targeting the improvement and retention of older employees, were identified [74,75,76,77]. They summarized 40 individual studies. Included studies differed regarding age; some studies included employees aged 40 years or 45 years and older whereas other studies did not define age-related inclusion criteria. Poscia et al. (2016) and Steenstra et al. (2017 recommended the implantation of multi-component organizational intervention programs involving changes of work-environment, physical training and psychosocial support to promote older employees working capacity, health and well-being [76,77]. However, Poscia et al. (2016) showed that learning in older employees in workplace intervention trainings differs from that in younger employees. Therefore, a multimodal approach is the method of choice [76].

### 3.6. Economic Effects

Economic analyses considered the following types of outcomes in ten reviews (77 single studies): absenteeism, productivity, net benefit and return on invest (ROI), compensated healthcare costs, other healthcare costs and intervention costs [24,78,79,80,81,82,83,84,85,86]. Low evidence levels are reported in four high-quality reviews and two moderate-quality reviews [24,80,81,82,83,84]. Furlan et al. (2012) reported economically successful interventions for the management of depression in the workplace [24]. The interventions consisted of cognitive-behavioral therapy and integrated supply management, as well as organizational components. Due to the heterogeneity of primary studies, Furlan et al. could not draw a general conclusion regarding economic effectiveness of mental health programs.

Hamberg-van Reenen, Proper and van den Berg (2012) [85] found a positive ROI of 302% of after two years. Carolan, Harris and Cavanagh (2017) [47] showed that web-based interventions resulted in increased productivity. Despite the low evidence levels, the authors of these reviews conclude that the evaluated interventions have great potential to be cost effective.

Opposing effects are reported for interventions to prevent musculoskeletal disorders on absenteeism and productivity by White et al. (2016) [84]. The economic impact of ergonomic interventions for caregivers was limited due to the small evidence base.

Overall, evidence to support sustainability of economic findings beyond one year was generally limited.

## 4. Discussion

### 4.1. Limitations and Strengths

The reviews included heterogeneous types of interventions which, again, varied across the studies. Variations in settings and study populations also limit the comparability. Therefore, the derivation of general recommendations for specific interventions or occupational groups is not possible.

Furthermore, publication bias was not addressed. Publication bias means that studies reporting positive effects are more likely to be published.

Some reviews focusing on the topic of mental health and musculoskeletal disorders identified the same primary studies, depending on the search strategy, inclusion criteria and execution period. If several reviews retrieved the same studies and reached the same conclusions, we evaluated information given on reported outcomes and populations to avoid over- or underestimating the strength of the evidence.

We found some opposing effects, for example regarding the impact of mental health programs combined with ergonomic programs. These opposing effects were not necessarily due to qualitative differences in the study designs. The ratio of studies reporting no effect to studies reporting statistically significant effects was rather equally distributed across the high-quality rated reviews.

Although this systematic review is relevant within the German context, it also has international meaning, as it is based on international research. 

### 4.2. Findings

Using the same search strategy and considering the main focus on the musculoskeletal system and mental health as well as older employees and economic evaluation, we found 74 references in a six-year period (2012–2017) compared with approximately 15 in an earlier period (2006–2012). There was a higher proportion of relevant high-quality studies as compared with the earlier review. No reference included reported negative effects as increased symptoms. However, job stress management training and workstation adjustment as single-component interventions had no effect on musculoskeletal outcomes. While we found evidence for the positive effect of multicomponent worksite programs, the findings are quite consistent with the earlier review and in agreement with other recent reviews that include workplace-based interventions [87,88,89].

Overall, there is not enough evidence from the scientific literature to recommend specific intervention or program. To this end, more high-quality evidence is needed. There was some consistency in the results, suggesting that workplace interventions such as exercising, trainings and educational programs and ergonomic desks and chairs can decrease pain and symptoms for employees who experience musculoskeletal disorders. However, the evidence is at best, moderate and current research still is limited. Our evidence synthesis provides support that workplace interventions can reduce absenteeism and associated costs. There was moderate evidence that cognitive-behavioral therapy, MBSR, and job stress management trainings, can significantly reduce stress; evidence for sustainability of these effects was insufficient or limited.

To date, guidance for practice to deal with the increasing numbers of older employees and work-related chronic conditions cannot be provided from systematic reviews.

### 4.3. General Recommendations and Practical Messages

The authors recommend that organizations continue to deliver workplace interventions to employees, since several interventions have been found to positively impact employees’ health. The findings indicate, that high quality implementation, including systematic evaluation and ongoing monitoring procedures lead to a higher efficacy. Special attention should be paid to the evaluation of interventions using appropriate designs to document the process and outcomes.

➢ Practitioners should consider implementing stretching exercise programs, vibration feedback on mouse use or workstation forearm supports in practices to prevent musculoskeletal disorders, if applicable to the work context.
➢ We recommend implementing workplace-based cognitive-behavioral and job-stress management programs to prevent and manage stress and mental disorders.
➢ Multi-component programs are to be preferred to single-component programs.
➢ When adopting interventions across diverse occupational groups and workplace settings, it is important to take into account both generic principles and to those principles that are specific to the given setting.
➢ A participative approach that engages employees, employers and management structures in communication and joint participation, appears to be an important success factor for the development and implementation of interventions for disease prevention and health promotion in the workplace.
➢ Prevention and health promotion interventions in the workplace often involve new approaches. Therefore, it can be necessary to make organizational changes.
➢ Still, this systematic review mainly identified individual focused interventions. Employers should significantly expand their programs on the organizational level.

## 5. Conclusions

The future development of workplace interventions needs to be based on sound knowledge and evidence. Therefore, appropriate evaluation frameworks and research methods capable of reflecting the complexity of the interventions have to be applied. This includes the evaluation of intermediary variables which refer to the implementation process and compliance aspects.

Economic analyses were conducted from a workplace perspective, as they primarily focused on wage replacement and healthcare costs. Future studies should address the societal and insurer perspective, including costs to the worker such as lost income, and lost time at work of family members due to caregiving activities. At the same time, additional structured activities focusing on epidemiology, surveillance and research can help to understand and improve employee health and derive recommendations for workplace health programs.

## Figures and Tables

**Table 1:** Distribution of reviews by quality rating and aims/outcome.

Aims/Outcome	Quality Rating			
	High *n* (%)	Moderate *n* (%)	Low & Very Low *n* (%)	N.A. *n* (%)
Mental disorders (*n* = 38)	7	12	17	2
	(18%)	(32%)	(45%)	(5%)
Musculosceletal disorders (*n* = 23)	6	9	7	1
	(26%)	(39%)	(30%)	(4%)
Older employees (*n* = 4)	0	4	0	0
	(0%)	(100%)	(0%)	(0%)
Economic impact (*n* = 10)	4	2	2	2
	(40%)	(20%)	(20%)	(20%)
